# Genetic Variants and Their Putative Effects on microRNA-Seed Sites: Characterization of the 3′ Untranslated Region of Genes Associated with Temperament

**DOI:** 10.3390/genes14051004

**Published:** 2023-04-28

**Authors:** Gilberto Ruiz-De-La-Cruz, Ana María Sifuentes-Rincón, Eduardo Casas, Francisco Alejandro Paredes-Sánchez, Gaspar Manuel Parra-Bracamonte, David G. Riley, George A. Perry, Thomas H. Welsh, Ronald D. Randel

**Affiliations:** 1 Laboratorio de Biotecnología Animal, Centro de Biotecnología Genómica, Instituto Politécnico Nacional, Reynosa 88710, Mexico; gruizd1800@alumno.ipn.mx (G.R.-D.-L.-C.); gparra@ipn.mx (G.M.P.-B.); 2 National Animal Disease Center, Agricultural Research Service, Unite States Department of Agriculture, Ames, IA 50010, USA; eduardo.casas@usda.gov; 3 Unidad Académica Multidisciplinaria Mante, Universidad Autónoma de Tamaulipas, Mante 89840, Mexico; faparedes@docentes.uat.edu.mx; 4 Department of Animal Science, Texas A&M University, College Station, TX 77843, USA; david.riley@ag.tamu.edu (D.G.R.);; 5 Texas A&M AgriLife Research, Overton, TX 75684, USA; george.perry@ag.tamu.edu (G.A.P.); ron.randel@ag.tamu.edu (R.D.R.)

**Keywords:** 3′UTR, SNP, candidate genes, cattle behavior, temperament, microRNA, seed site

## Abstract

The 3′ untranslated region has an important role in gene regulation through microRNAs, and it has been estimated that microRNAs regulate up to 50% of coding genes in mammals. With the aim of allelic variant identification of 3′ untranslated region microRNA seed sites, the 3′ untranslated region was searched for seed sites of four temperament-associated genes (*CACNG4*, *EXOC4*, *NRXN3*, and *SLC9A4*). The microRNA seed sites were predicted in the four genes, and the *CACNG4* gene had the greatest number with 12 predictions. To search for variants affecting the predicted microRNA seed sites, the four 3′ untranslated regions were re-sequenced in a Brahman cattle population. Eleven single nucleotide polymorphisms were identified in the *CACNG4*, and eleven in the *SLC9A4*. Rs522648682:T>G of the *CACNG4* gene was located at the predicted seed site for *bta-miR-191*. Rs522648682:T>G evidenced an association with both exit velocity (*p* = 0.0054) and temperament score (*p* = 0.0097). The genotype TT had a lower mean exit velocity (2.93 ± 0.4 m/s) compared with the TG and GG genotypes (3.91 ± 0.46 m/s and 3.67 ± 0.46 m/s, respectively). The allele associated with the temperamental phenotype antagonizes the seed site, disrupting the *bta-miR-191* recognition. The G allele of *CACNG4*-rs522648682 has the potential to influence bovine temperament through a mechanism associated with unspecific recognition of *bta-miR-191*.

## 1. Introduction

Temperament in cattle is an important trait observed as a response to handling and human-facing activities [1]. There is a growing list of candidate genes associated with temperament traits [2,3], and their complex genetic architecture still needs to be elucidated. Most genetic variations in candidate genes for temperament are based on Single Nucleotide Polymorphisms (SNPs). These markers are relevant due to their functional implications. For instance, SNPs in coding regions can have amino acid substitution with an effect on protein structure [4], whereas those located in regions such as enhancer, intron, and untranslated regions could be involved in recognition sequences such as transcription factors, splicing, regulatory regions, and sequences of epigenetic modulation [5].

The 3′ untranslated region (3′UTR) particularly has an important role in gene regulation through microRNAs (miRNAs). The miRNAs are processed from stem-loop regions of longer RNA transcripts that are ~22 nucleotides (nt) in length [6], and they have a cis-regulatory role in 3′UTR regions [7]. It has been estimated that miRNAs regulate up to 50% of coding genes in mammals [8]. Regulation by miRNAs occurs through recognition of a heptamer of 2–8 nucleotides known as the seed region that binds to messenger RNA (mRNA) at a complementary site located in the 3′UTR sequence. Therefore, the 3′UTR seed site is highly conserved; a change in this site could antagonize the complementary binding of the miRNA-mRNA to a partial binding, with an effect on expression or repression [7].

It has been widely documented that the SNPs could modify the seed site, allowing for changes in gene expression in humans and domestic animal species. Orthologous genes of serotonin receptors associated with temperament have been susceptible to repression by a polymorphism in the 3′UTR of mRNA, affecting aggressive behavior [9]. In contrast, the repression loss for miR-96 by the allelic change in the seed site of the *HTR1B* gene (A/G) caused a phenotypic change in the aggressiveness of mice [10]. The association of variants in genes of subunits of voltage-gated calcium channels for the treatment of schizophrenia have been identified, and SNP genotypes in the 3′UTR region of *CACNA1B* gene were associated with an increase in symptoms and allowed for the explanation that the regulation was potentially mediated the modification of the seed site [11]. From the growing list of candidate genes associated with cattle temperament, Paredes-Sánchez et al. [12] found an important association of temperament traits with five intronic SNPs located in the neurexin-3 (NRXN3), calcium voltage-gated channel auxiliary subunit gamma-4 (CACNG4), exocyst complex component-4 (EXOC4), and solute carrier family 9 member A4 (SLC9A4) genes. These candidate genes were implicated in different biological pathways related to the expression of behaviors as a response to stress and fear, supporting their possible role in cattle temperament [12], but their precise implications for gene expression and or regulation requires more study.

Due to the potentially important role of variations in the 3′UTR, this targeted study characterized the 3′UTR in the four candidate genes (*CACNG4*, *EXOC4*, *NRXN3*, and *SLC9A4*). As a first step, bioinformatic analysis was achieved to detect potential seed sites, and as a second step resequencing each 3′UTR from each gene was accomplished by searching for genetic variation and its effect on regions important for miRNA interactions.

## 2. Materials and Methods

### 2.1. Biological Material

The animal data came from a reference population with a pedigree structure, including 242 dams and 12 sires. One hundred sixty-four calves were selected from a population of three hundred fifty-five Brahman calves (one hundred eighty-one males and one hundred seventy-four females) born between 2018 and 2020 at the Texas A&M AgriLife Research and Extension Center in Overton, TX. The selected calves were those with a single dam (*n* = 148) and dam half-siblings (*n* = 16). Birth year distribution was: 2018 (*n* = 91, 41 males and 50 females); 2019 (*n* = 40, 20 males and 20 females); and 2020 (*n* = 33, 15 males and 10 females). All calves had data recorded for temperament traits. The temperament tests applied were: exit velocity (EV), an objective test that measures the speed of an animal when traveling 1.8 m after release from a holding chute, measured using an infrared sensor (FarmTek, Inc., North Wylie, TX, USA); pen score (PS), a subjective measurement test through the visual evaluation of animal behavior, assigning scale values where 1 represents a docile animal and 5 points to an aggressive one; temperament score was calculated as the average of the EV and PS values: [TS = (EV + PS)/2] [12,13]. DNA was obtained from ear notches, taken during temperament evaluation, and stored at −80 °C. DNA extraction was performed with the commercial Genelute Mammalian Genomic DNA kit (Cat. G1N350, Sigma-Aldrich Co. LLC, St. Louis, MO, USA).

All practices complied with the Guide for the Care and Use of Agricultural Animals in Research and Teaching 2010 and were approved by the Texas A&M University Animal Care and Use Committee AUP 2002-315.

### 2.2. In Silico Analysis to Locate Seed Sites for miRNAs and their Potential Disrupting of SNPs

The 3′UTR analysis region from *CACNG4*, *EXOC4*, *NRXN3*, and *SLC9A4* genes was determined using as a reference the mRNA sequence obtained from the National Center for Biotechnology Information (NCBI) public database (https://www.ncbi.nlm.nih.gov/ (accessed on 15 March 2022)). Using the ARS-UCD 1.3 version of the bovine genome and taking the last position of the final exon defined from the termination codon (TGA) of mRNA, using the Genome Data Viewer tool (https://www.ncbi.nlm.nih.gov/genome/gdv/ (accessed on 15 March 2022)). The reference sequences were the *Bos taurus* (L., 1758) genome, so we searched the *Bos indicus* (L., 1758) genome (version Bos_indicus_1.0) sequences for alignment with the Clustal Omega (https://www.ebi.ac.uk/Tools/msa/clustalo/ (accessed on 18 March 2022)) tool, described by Madeira et al. [14] to ensure a percentage of identity through genomes.

The search of miRNAs homologous sites was carried out in the miRBase v.22.1 database (https://mirbase.org/ (accessed on 23 March 2022)) [15] by taking the previously defined 3′UTR and uploading it into the search engine, specifying for the mature sequence “Mature miRNAs” using the BLASTN method, which allows for searching for homologous miRNAs in long sequences, up to 1000 base pairs (bp), using the “*Bos taurus*” taxon. The sequences longer than 1000 bp were overlapped with fragments of 250 bp.

Once the miRNA families were identified, the ENSEMBL database (http://ensembl.org/ (accessed on 25 March 2022)) was searched for SNPs, using a search engine for “Cow” and the gene name (e.g., *CACNG4*). Next, a search was made in the “Genetic Variation” tab, filtering the results by SNP class variations and “Consequences: 3 prime UTR variant”. The sequences were aligned “Pairwise” between miRNAs and 3′UTR with the GenScript tool (https://www.genscript.com/sms2/pairwise_align_dna.html (accessed 30 March 2022)); a perfect complementarity between sequences was sought to have a reliable prediction, locating the seed site and positioning the SNPs.

If an SNP in the seed site was found, the gene sequence with the allelic change was searched in miRBase to determine if there was a loss of homology recognition. We examined for evolutionary conservation between the miRNA and the gene sequence for miRNA search in the microRNAviewer matrix (https://people.csail.mit.edu/akiezun/microRNAviewer/ (accessed on 1 April 2022)) that shows a global view of homologous miRNA genes [16], whereas for the mRNA conservation the 3′UTR of genes was searched among *B*. *taurus*, *B*. *indicus*, *B*. *mutus*, *Ovis aries*, *Capra hircus*, *Sus scrofa*, *Equus caballus*, and *Homo sapiens*.

The binding of miRNA-mRNA was evaluated for target site accessibility and free energy through mRNA sequence (3′UTR). The RNAhybrid (https://bibiserv.cebitec.uni-bielefeld.de/rnahybrid/ (accessed on 10 October 2022)) [17] tool finds the minimum free energy (MFE) hybridization between RNA sequences, setting the stringency value at <−20 kcal/mol [18]. To calculate the allelic effect, a change in MFE (ΔMFE) between both variants was calculated with MFE_reference_ − MFE_alternative_ [19].

### 2.3. Searching for SNPs in 3′UTR and Their Effect on Brahman Cattle Temperament Traits

Samples of 65 calves of the Brahman breed were used as the discovery population. It included animals with docile and temperamental scores selected according to categorical scores for docility, with EV ranges of 0.16–1.82 m/s and 0.4–1.56 m/s for females and males, respectively, and temperamental ranges of 3.13–7.66 m/s and 3.05–10.83 for females and males, respectively [12]. Deoxyribonucleic acid (DNA) from these samples were used to amplify the 3′UTR region of *CACNG4*, *EXOC4*, *NRXN3*, and *SLC9A4* genes using specific primers (Table 1). All of the polymerase chain reaction (PCR) products were accomplished using the Nextera Flex protocol from Illumina (Part #15044223 Rev. B).

Results of 3′UTR sequencing were analyzed through the Genome Analysis Toolkit (GATK.3.7) [20], a program group used for variant discovery and genotyping [21]. Reads were aligned with the bovine genome (https://hgdownload.soe.ucsc.edu/goldenPath/bosTau9/bigZips/ (accessed on 8 June 2022)) for total BWA (Burrows–Wheeler alienator) [22], with exact matches that cannot be extended in either direction through Maximal Exact Match for quality control. Once the alignment was performed, step data cleaning was followed by identifying and deleting repeated reads and the selection of major reads with Picar, generating BAI and BAM files. Finally, the outputs were submitted to HaplotypeCaller of GATK to identify variants, extract and filter SNPs, thus generating an output file containing the position of variations, alleles, and quality parameters.

### 2.4. Effect of CACNG4-3′UTR SNPs on Temperament Traits

DNA samples from 164 Brahman calves were used for SNP genotyping. Samples were amplified with primers (5′-CCATTATCTCCTCTTGGGAA-3′, and 3′-ACCCTTTCTGTGAGCAG-5′) flanking the region of *CACNG4*-3′UTR that includes the seed site with the rs522648682:T>G SNP. The allelic assignment was achieved once the products were sequenced using SimpleSeq™ (Eurofins Genomics, LLC, Louisville, Kentucky, USA), and the allelic frequency analysis was determined using Cervus 3.0.7 software [23].

### 2.5. Statistical Association Analysis

A Shapiro–Wilk test was performed on exit velocity, pen score, and temperament score traits, to verify normality. Posteriorly, to estimate the effects of rs522648682:T>G, rs483247086:A>C, and rs109514077:C>T genotypes on the studied temperament traits, a statistical model was fitted using the MIXED procedure in SAS OD for Academics (SAS Institute Inc., Cary, NC, USA) as follows:

$$Y_{ijklm}=\mu +S_{i}+D_{j}+G_{k}+X_{l}+Y_{m}+\beta_{1}d_{j}+\beta_{2}d_{j}{}^{2}+\varepsilon_{ijklm}$$

where: *Y_ijk_* = temperament traits (EV, PS, and TS), *μ* = general mean, *S_i_* = random effect common to the progeny of the *i*-th sire, *D_j_* = random effect common to the progeny of the *j*-th dam, *G_k_* = fixed effect of *k*-th genotype of loci, *X_l_* = fixed effect of *l*-th sex of animal, *Y_m_* = fixed effect of *m*-th year of birth, *β*_1_*d_j_* = fixed effect of linear covariate for age years of *j*-th dam, *β*_2_*d_j_*^2^ = fixed effect of quadratic covariate for age years of *j*-th dam, and ε*_ijkm_* = random error.

Least-squares means for the genotypes of the studied loci were estimated and compared using a PDIFF statement considering an alpha = 0.05.

## 3. Results

### 3.1. In Silico Analysis of Seed Site and SNP Variants in 3′UTR Sequences

A total of 27 seed sites for miRNAs distributed in the four target genes were found. The CACNG4 and NRXN3 had twelve and eleven predicted seed sites, while EXOC4 and SLC9A4 had one and three seed sites, respectively (Table 2).

After the four studied genes were interrogated for database-reported SNPs in their 3ÚTR region, we found that only the CACNG4 gene had 274 reported SNPs, and from those only the transversion T>G (rs522648682) was located at a previously identified miRNA seed site, the bta-miR-191.

**Figure 1:**
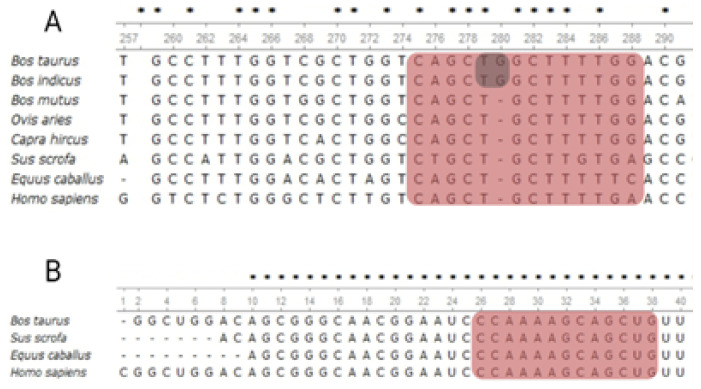
Characterization of miR-191 as a putative seed site in the 3′UTR-CACNG4. (**A**) alignment of 3′UTR-CACNG4; the red background displays the putative seed site sequence for miR-191. The SNP is shown in the grey background. The percentage of AT nucleotides for the CACNG4 seed site was 47.67% without considering the SNP, whereas the flanking sequence (30 nucleotides) was 46.67%. (**B**) miR-191 gene alignment. The alignment shows a hairpin sequence of the positive strand, with a red background for the seed site’s recognition sequence; recognition by miRNA would correspond to an arm 3′ of the miRNA strand (3p). * Conserved position among all the sequences in the alignment.

The prediction analysis shows that the seed site perfectly complements the bta-miR-191 presence in the 3′UTR-CACNG4 gene, whereas the allelic change T>G induces the loss of bta-miR-191 recognition (Figure 1).

A homology analysis revealed the seed site for miR-191 located in the bovine CACNG4 gene is conserved in different mammalian species such as sheep, goat, horse, yak, and humans (Figure 1A). The complete seed site is conserved among the Bovidae family species. The homology of the miR-191 gene was also high between the included species, with a 1.0–0.94 conservation value reported in the microRNAviewer matrix (Figure 1B). In addition to the conservation value, the evaluation of binding between mRNA-miRNA shows that the allele T had a MFE = −33.0 kcal/mol, whereas the G allele had a MFE = −28.9 kcal/mol, with a ΔMFE value of 4.1 kcal/mol. Negative ΔMFE displays increased binding, whereas a positive value indicates decreased binding in the variant allele. These results support the importance of this region as a miRNA target recognition in the bovine CACNG4 gene.

### 3.2. Search for Variants in 3′UTR of the Four Temperament Candidate Genes in Brahman Cattle

The resequencing of the 3′UTR region in the four studied genes shows that the EXOC4 and NRXN3 genes did not have SNP variants, while a total of twenty-two variants were located in the CACNG4 (n = 11) and SLC9A4 (n = 11) genes (Table 3).

None of the eleven SNPs in the SLC9A4 gene were located at any of the in silico predicted seed site positions; however, the analysis of the CACNG4 SNPs allowed for confirmation in the studied Brahman population, with the presence of SNP rs522648682:T>G previously identified in the in silico analysis as a potential SNP responsible for disrupting a seed site in the 3′UTR of the CACNG4 gene (Figure 1A).

### 3.3. Association of the SNP rs522648682 Located at 3′UTR of CACNG4 Gene with Temperament Traits in Brahman Cattle

The allelic frequencies in the genotyped Brahman cattle show that only the rs522648682:T>G located at the seed site for bta-miR-191 on the CACNG4 gene was polymorphic (T = 0.3476, G = 0.6524) according to the minor allele frequency >10%.

The results for the association analysis of rs522648682:T>G with three temperament traits (EV, PS, and TS) are shown in Table 4. A significant effect for EV and TS (*p* < 0.05) was observes; the carriers of the GG genotype had an EV value of 0.74 m/s and a TS value of 1.23 SD, which was greater than the TT genotype. No significant differences were observed between the heterozygous genotype (TG) and GG homozygous genotypes.

## 4. Discussion

The genotypes of rs522648682-CACNG4 were associated with EV and TS. The genotypes with alternative allele G favored the highest temperamental values, and in silico analysis shows a lost recognition and unstable hybridization for bta-miRNA-191. In contrast, a homozygous genotype (TT) with a reference allele shows docility and stable hybridization in silico between miRNA-mRNA. The GG and TG genotype differences between both temperament tests (0.24 m/s EV and 0.008 SD TS) were similar, whereas among genotypes with significantly different means (TT vs. TG, GG), animals with the G allele were approximately 25% faster in terms of EV. Additionally, the population had a Hardy–Weinberg equilibrium for the rs522648682, supporting the population size effective genotyping; however, the sample number for the association analysis is still limiting for the use of rs522648682-CACNG4 as a marker to assist in cattle temperament selection.

Our in silico search found seed sites for potential miRNA in the four temperament candidate genes analyzed. To our knowledge, none of the predicted miRNAs have been shown to influence the gene expression patterns or temperament traits.

The interaction with miRNAs plays a crucial role in the post-transcriptional regulation of gene expression [24]. Therefore, the presence of SNPs in the seed site could create illegitimate sites, interfere with the recognition site, and promote gene expression change [25]. Here, we found in silico and experimental evidence that the *miR-191* seed site could be antagonized by the rs522648682 SNP located at the 3′UTR of *CACNG4*. The seed site comprises a heptamer of 2–8 highly conservated nucleotides for miRNA recognition [6], and it can have a total or partial complementarity between miRNA and mRNA [26].

The rs522648682 was located on position 5, disrupting the architecture of the seed site through allele change. The heptamer had conservated sequences among *Bovidae*, sheep, goat, horse, and humans. Additionally, the *miR-191* gene has conservation and homology between humans, bovine, and horse, according to the matrix comparison performed using microRNAviewer and supported by the experimental evidence of miRBase. However, seed site matches are insufficient as evidence for a putative repression effect, and some additional attributes increase the effectiveness of functional site prediction. Grimson et al. [27] proposed a scoring scheme with a high AU percentage of around 30 nucleotides upstream and downstream from the seed site, while Long et al. [28] found that seed sites with regulatory activity have between 47.8–41.2% AU composition; these last results coincide with the values observed in our study (Figure 1A).

It has been proposed that duplex analysis also supports the prediction of binding between miRNA-mRNA sequences [29]. The RNA duplex is more stable and thermodynamically stronger when MFE is lower, setting an MFE value threshold for the seed site search at MFE ≤ 20 [18]. In addition to the structural recognition of 13 nucleotides between 3′UTR-*CACNG4* and *bta-miR-191*, our hybridization analysis found positive differences when T was replaced by G (ΔMFE = 4.1 kcal/mol). The reference and alternative allele MFE value difference affected hybridization [19], with a positive value increase in MFE having the potential to form a weak RNA duplex [30].

According to the literature, three types of duplex structures can be presented: the canonical with perfect 5′ to 3′ complementarity, the dominant seed sites type with perfect complementarity at the 5′ of the seed site but poor complementarity at the 3′, and compensatory sites with a mismatch at the 5′ end compensated by a complementarity at the 3′ [31]. The SNP could impact the duplex structure type, since the seed site’s reference allele T generates a canonical type and the alternative G is a compensatory site. Non-canonical sites generally do not lead to mRNA modulation by miRNA binding [32,33].

In humans, miR-191 has been extensively studied, and due to its role in the regulation of many diseases, especially different cancer types, it is considered as an important candidate biomarker [34]. Interestingly, *miR-191-5p* is part of a group of unique circulating miRNAs that are able to distinguish with high precision an Alzheimer’s disease patient from controls [35]; thus, it is also considered as a potential biomarker. In cattle, *bta-miR-191* has not been reported as a regulator of *CACNG4*. Nevertheless, *bta-miR-191* has been reported via expression profile analysis in bovine blood serum and plasma [36]. In silico approaches and reporter system information have shown that *miR-191* directly represses brain-derived neurotrophic factor (BDNF) through binding in 3′UTR in a mouse model [37].

Modulation changes of the *CACNG4* gene could contribute to temperament variation since voltage-gated channel genes influence bovine temperament and behavioral activities [38]. Genome-wide association studies in bovine evaluated SNPs for temperament and tenderness traits and found temperament associations in genes with transport, complexes, and activity voltage-gated channels for sodium ions [38]. Voltage-gated channels maintain depolarization, allowing one impulse to be executed [39]. Voltage-gated ion channels allow ions to pass through membranes, forming part of cellular communication in neurons [40]. The nerve impulse could be a possibility for temperament variation studies because it uses voltage-gated channels for faster conduction; it is also an evolutionary advance through subunit unions to form selective channels such as calcium and sodium [41].

Some SNPs located at some calcium channel genes have been reported. In humans, a genotype of rs2229949 on 3′UTR of *CACNA1B* was associated with adverse behavior symptomatology, predicting that allelic change abolishes a seed site for regulatory miRNA [11].

Additionally, some functions of calcium channels influence physiological responses such as secretion, contraction, and gene transcription. Therefore, mutations can dysregulate their functions and produce a behavior disorder, as the human model indicated by showed neuropsychiatric disease [42]. The genes of subunit gamma are reported to have encoded transmembrane regulators of glutamate receptors [43]. The glutamate receptors (AMPAR) initiate the excitatory neurotransmission and mediate ligand-gated ion channels required to propagate the electrical signal in the brain [44]. AMPAR can increase the reliability of synaptic signals and develop memory formation and learning [44].

Genome association studies have discovered epilepsy disease susceptibility to variations in the *CACNA1A* gene [45]. Additionally, the GABA_A_ receptor gamma-3 (*GABRG3*) has a suggestive association with aggressiveness temperament in bovines [46]. Therefore, an imbalance in the propagation of neurotransmitters stimulated by a voltage-gated calcium channel switch that affects the glutamate pathway through AMPA receptors and enriched by changes in GABA_A_ receptors could trigger a variation in regulation, which contributes to the difference in temperament phenotypes, given that it has the potential for activity in a pathway such as excitatory neurotransmission.

## 5. Conclusions

The 3′UTR of temperament candidate genes is a structural region harboring putative complementary sites for miRNAs with the potential to have an effect on gene expression and therefore aid in the elucidation of complex functional traits. Allelic variations in the 3′UTR could alter the recognition of miRNAs seed sites as it was shown for the CACNG- rs522648682:T>G SNP located at the bta-miR-191 seed site. Further studies are needed to confirm the role of the bta-miR-191 seed site in CACNG4 gene expression and a broadened validation assessment could confirm the SNP rs522648682 as an additional DNA marker for cattle temperament trait selection.

## Figures and Tables

**Table 1:** Primers designed for the amplification of the 3′UTR sequence.

GENE		PRIMER	POS	T_M_	T_M__S	AMPLICON (bp)
*CACNG4*	F	TCTTCCAGCAGGACCTCAGC	63112792:63112811	58	58 °C	2195
	R	CCCTTCCCTGCCCAACATCA	63114967:63114986	59		
*EXOC4*	F	TCATCTGCGAGCAGGCA	97744687:97744703	56	54 °C	1675
	R	CCTTCCACAGACTCTCCTGG	97746342:97746361	56		
*NRXN3*	F	GCTCAAGGAGAAGCCAC	91099821:91099837	52	52 °C	2708
	R	TAGTTCCCATGAACATGGTC	91102509:91102528	51		
*SLC9A4*	F	TGTCCCACAGCCCTTTG	7296345:7296361	54	54 °C	1379
	R	TGTGGCTGGGTTTTCTCA	7297706:7297723	53		

GENE: gene names; PRIMER: sequence of primers, F as forward, and R as reverse; POS: genome position amplification; T_M_: melting temperature; T_M__S: average melting temperature; AMPLICON (bp): size in base pairs of polymerase chain reaction products amplified with primers.

**Table 2:** Putative seed sites sequences for recognition between miRNAs-mRNA located in candidate genes for temperament.

GENE	miRNA	Seed Site	Strand
*CACNG4*	*bta-miR-10173-5p*	AGGAAGUGAGGAAGGAGCCAGA	+
	*bta-miR-12046*	UUCUGCUCCCUGCCCUGUAG	−
	*bta-miR-1225-3p*	CCGAGCCCCUGUGCCGCCCCCAG	+
	*bta-miR-191*	CAACGGAAUCCCAAAAGCAGCUG	−
	*bta-miR-2284d*	AAAAAGUUCGUUAGGGUUUUUC	−
	*bta-miR-2374*	UUGGGGCUGGGGAGAGGCGGG	−
	*bta-miR-2442*	AGAGCAGGGGCUGUGGGCUGCA	+
	*bta-miR-2882*	AGCCCGGGCCCCUCCCCUG	+
	*bta-miR-2882*	GCCCGGGCCCCUCCCCU	+
	*bta-miR-376e*	AACAUAGAGGAAAAUCCACAUU	+
	*bta-miR-769*	UGAGACCUCCGGGUUCUGAGCU	+
	*bta-miR-99a-3p*	CCCAUAGAAGCGAGC	−
*EXOC4*	*bta-miR-11981*	CAGGGCGGGAACGGGCUGCGGGA	+
*NRXN3*	*bta-miR-10175-5p*	UGGAGAGAACAGGUGGCUUU	+
	*bta-miR-1603*	GUGGUUUGUUUUGUGUUUUU	−
	*bta-miR-1777a*	UGGGGGCGGUGGGGGGCGGG	−
	*bta-miR-1777b*	GGGGGCGGUGGGGGGCGGGG	−
	*bta-miR-2285bp*	AAAACCAGAACGAACUUUGUGU	−
	*bta-miR-2285br*	AAAACCUGAAUGAACUUUCUGU	−
	*bta-miR-2293*	UGAUUUUGUUGUUUUGUAUU	−
	*bta-miR-2305*	CGGGGGUGGCGGGGAGGGGG	−
	*bta-miR-2393*	UAGAUUUUUUGUUUUCUUUU	−
	*bta-miR-2421*	UAUUUUUUUGUUUCGUGUUU	−
	*bta-miR-2444*	UUUGUGUUGUUUUUUGUUUU	−
*SLC9A4*	*bta-miR-1256*	AGGCAUUGACUUCUCUCUAGAU	+
	*bta-miR-2381*	CAGGCUGCUCUGUGCUUGGCU	+
	*bta-miR-2895*	CCUGCUGAUCUCACAUUAAUUCA	−

GENE: gene names; miRNA: microRNA name; seed site: sequence of recognition between microRNA and messenger RNA; strand: sequence origin strand of microRNA.

**Table 3:** Identification of SNP variants in temperament candidate genes in Brahman cattle.

GENE	SNP ID	POS	REF	ALT
*SLC9A4*	rs474342472	7296410	G	T
	rs210547017	7296519	A	C
	rs43657583	7296536	C	A
	rs43657582	7296598	T	C
	rs381837194	7296603	T	C
	rs211375703	7296607	T	C
	rs43657581	7296702	T	A
	rs383852181	7296963	G	A
	rs207631524	7296967	G	A
	rs43657580	7297019	A	G
	rs43657579	7297241	A	C
*CACNG4*	rs109550544	63112976	C	G
	rs721644985	63113133	A	C
	rs522648682	63113244	T	G
	rs483247086	63113258	A	C
	rs109514077	63113339	C	T
	rs527101469	63113346	T	C
	rs516574957	63114300	C	T
	rs520252985	63114406	C	T
	rs720236805	63114561	C	T
	rs472138682	63114815	A	T
	rs724115129	63114920	C	T

SNP ID: identificatory of SNP in Ensemble database; POS: genome position; REF: reference allele; ALT: alternative allele.

**Table 4:** Least-squares means and standard errors of the effect for rs522648682 on temperament traits.

SNP	*n*	*p*-Value	Genotype		
rs522648682			TT	TG	GG
EV	164	0.0054	2.93 ± 0.47 ^a^	3.91 ± 0.468 ^b^	3.67 ± 0.466 ^b^
PS	164	0.1913	1.96 ± 0.43	2.29 ± 0.44	2.35 ± 0.44
TS	164	0.0097	2.3 ± 0.38 ^a^	2.95 ± 0.38 ^b^	2.87 ± 0.383 ^b^

^a,b^ Means with different superscripts within columns are statistically different (*p* < 0.05); SNP: identificatory of SNP in Ensemble database; *n*: number of samples; *p*-value: significance value for the statistical test; genotype: a combination of alleles according to the SNP alternative; EV: exit velocity; PS: pen score; TS: temperament score.

## Data Availability

Data available on request from the authors.
